# Supplementary material for: Whole genome sequencing of Plasmodium vivax isolates reveals frequent sequence and structural polymorphisms in erythrocyte binding genes
Source: PLoS Negl Trop Dis. 2020 Oct 12;14(10):e0008234. doi: 10.1371/journal.pntd.0008234 (PMC7581005; doi:10.1371/journal.pntd.0008234)
Supplement: S1 Table — (DOCX) [file pntd.0008234.s001.docx]

**Supplementary Table 1.** Distribution of SNP variants in the 43 *P. vivax* erythrocyte binding gene candidates among the 44 Ethiopian genomes.

| Chromosome | Gene | Gene ID  (PlasmoDB) | Gene Description | Total number of SNP variants | Number of nonsynonymous SNPs (%) | Number of synonymous SNPs (%) |
| --- | --- | --- | --- | --- | --- | --- |
| 1 | DBP2 | PVP01_0102300 | Duffy binding protein 2/EBP | 49 | 13 (26.5%) | 36 (73.5%) |
| 2 | TRAG2 | PVP01_0202200 | tryptophan-rich protein | 9 | 2 (22.2%) | 7 (77.8%) |
| 3 | RON1 | PVP01_0305300 | rhoptry neck protein 1 | 21 | 4 (19.1%) | 17 (80.9%) |
| 4 | TRAG3 | PVP01_0404200 | tryptophan-rich protein | 114 | 7 (6.1%) | 107 (93.9%) |
| 4 | MSP5 | PVP01_0418400 | merozoite surface protein 5 | 10 | 9 (90%) | 1 (10%) |
| 4 | MSP4 | PVP01_0418300 | merozoite surface protein 4 | 5 | 3 (60%) | 2 (40%) |
| 5 | TRAG4 | PVP01_0503400 | tryptophan-rich protein | 65 | 1 (1.5%) | 64 (98.5%) |
| 5 | TRAG6 | PVP01_0503700 | tryptophan-rich protein | 10 | 3 (30%) | 7 (70%) |
| 5 | TRAG7 | PVP01_0504200 | tryptophan-rich protein | 10 | 3 (30%) | 7 (70%) |
| 5 | GAMA | PVP01_0505600 | GPI-anchored micronemal antigen | 5 | 0 (0%) | 5(100%) |
| 5 | TRAG38 | PVP01_0503600 | tryptophan-rich protein | 3 | 3 (100%) | 0 (0%) |
| 6 | DBP1 | PVP01_0623800 | Duffy binding protein | 13 | 12 (92.3%) | 1 (7.7%) |
| 6 | ebp2 | PVP01_0613400 | rRNA-processing protein EBP2, putative | 2 | 1 (50%) | 1 (50%) |
| 7 | MSP1 | PVP01_0728900 | merozoite surface protein 1 | 251 | 143 (57%) | 108 (43%) |
| 7 | RBP1a | PVP01_0701200 | reticulocyte binding protein 1a | 44 | 30 (68.2%) | 14 (31.8%) |
| 7 | TRAG35 | PVP01_0700800 | tryptophan-rich protein | 13 | 1 (7.7%) | 12 (92.3%) |
| 7 | RBP1b | PVP01_0701100 | reticulocyte binding protein 1b | 23 | 16 (69.6%) | 7 (30.4%) |
| 7 | RhopH3 | PVP01_0703800 | high molecular weight rhoptry protein 3, putative | 21 | 2 (9.5%) | 19 (90.5%) |
| 7 | MSP1P | PVP01_0728800 | merozoite surface protein 1 paralog | 17 | 11 (64.7%) | 6 (35.3%) |
| 7 | TRAG34 | PVP01_0700700 | tryptophan-rich protein | 4 | 2 (50%) | 2 (50%) |
| 8 | TRAG14 | PVP01_0801800 | tryptophan-rich protein | 33 | 6 (18.2%) | 27 (81.8%) |
| 8 | TRA2B | PVP01_0802200 | transformer-2 protein homolog beta, putative | 9 | 0 (0%) | 9 (100%) |
| 8 | MA | PVP01_0824100 | microneme associated antigen, putative | 9 | 5 (55.6%) | 4 (44.4%) |
| 8 | RA | PVP01_0812300 | rhoptry associated adhesin, putative | 6 | 2 (33.3%) | 4 (66.7%) |
| 9 | AMA1 | PVP01_0934200 | apical membrane antigen 1 | 18 | 12 (66.7%) | 6 (33.3%) |
| 9 | RON4 | PVP01_0916600 | rhoptry neck protein 4 | 11 | 0 (0%) | 11 (100%) |
| 9 | Trx-mero | PVP01_0905200 | thioredoxin-like mero protein, putative | 18 | 12 (66.7%) | 6 (33.3%) |
| 10 | MSP3.5 | PVP01_1031400 | merozoite surface protein 3 | 458 | 219 (47.8%) | 239 (52.2%) |
| 10 | MSP3.9 | PVP01_1031200 | merozoite surface protein 3 | 362 | 203 (56.1%) | 159 (43.9%) |
| 10 | MSP3.8 | PVP01_1031300 | merozoite surface protein 3 | 310 | 188 (60.7%) | 122 (39.4%) |
| 10 | MSP3.10 | PVP01_1031000 | merozoite surface protein 3 | 137 | 71 (51.8%) | 66 (48.2%) |
| 10 | MSP3.11 | PVP01_1030900 | merozoite surface protein 3 | 49 | 3 (6.1%) | 46 (93.9%) |
| 10 | MSP3G | PVP01_1031100 | merozoite surface protein 3 | 14 | 7 (50%) | 7 (50%) |
| 11 | TRAG19 | PVP01_1101400 | tryptophan-rich protein | 15 | 4 (26.7%) | 11 (73.3%) |
| 11 | MSP10 | PVP01_1129100 | merozoite surface protein 10, putative | 5 | 4 (80%) | 1 (20%) |
| 12 | RON2 | PVP01_1255000 | rhoptry neck protein 2 | 33 | 11 (33.3%) | 22 (66.7%) |
| 12 | TRAG20 | PVP01_1201800 | tryptophan-rich protein | 14 | 4 (28.6%) | 10 (71.4%) |
| 14 | RBP2a | PVP01_1402400 | reticulocyte binding protein 2a | 35 | 20 (57.1%) | 15 (42.9%) |
| 14 | TRAG22 | PVP01_1469800 | tryptophan-rich protein | 30 | 7 (23.3%) | 23 (76.7%) |
| 14 | TRAG23 | PVP01_1469900 | tryptophan-rich protein | 17 | 4 (23.5%) | 13 (76.5%) |
| 14 | MSP9 | PVP01_1446800 | merozoite surface protein 9 | 15 | 6 (40%) | 9 (60%) |
| 14 | TRAG24 | PVP01_1470100 | tryptophan-rich protein | 11 | 1 (9.1%) | 10 (90.9%) |
| 14 | TRAG21 | PVP01_1401800 | tryptophan-rich protein | 1 | 0 (0%) | 1 (100%) |
| **Total** |  |  |  | **2299** | **1055 (45.9%)** | **1244 (54.1%)** |
