# Supplementary material for: Whole genome sequencing of Plasmodium vivax isolates reveals frequent sequence and structural polymorphisms in erythrocyte binding genes
Source: PLoS Negl Trop Dis. 2020 Oct 12;14(10):e0008234. doi: 10.1371/journal.pntd.0008234 (PMC7581005; doi:10.1371/journal.pntd.0008234)
Supplement: S2 Table — (DOCX) [file pntd.0008234.s002.docx]

**Supplementary Table 2.** Distribution of single nucleotide polymorphism (SNP) variants across *P. vivax* chromosomes of the 44 Ethiopian genomes.

| Chromosome | Total number of SNP variants | Number of nonsynonymous SNPs (%) | Number of synonymous SNPs (%) |
| --- | --- | --- | --- |
| 1 | 10627 | 2761 (26%) | 7866 (74%) |
| 2 | 1912 | 586 (30.6%) | 1326 (69.4%) |
| 3 | 6228 | 1566 (25.1%) | 4662 (74.9%) |
| 4 | 11354 | 2452 (21.6%) | 8902 (78.4%) |
| 5 | 8347 | 1849 (22.2%) | 6498 (77.8%) |
| 6 | 2977 | 898 (30.2%) | 2079 (69.8%) |
| 7 | 4779 | 1332 (27.9%) | 3447 (72.1%) |
| 8 | 5777 | 1640 (28.4%) | 4137 (71.6%) |
| 9 | 24007 | 2754 (11.5%) | 21253 (88.5%) |
| 10 | 16852 | 3956 (23.5%) | 12896 (76.5%) |
| 11 | 6993 | 1958 (28%) | 5035 (72%) |
| 12 | 9646 | 2575 (26.7%) | 7071 (73.3%) |
| 13 | 5666 | 1385 (24.4%) | 4281 (75.6%) |
| 14 | 8546 | 2406 (28.2%) | 6140 (71.8%) |
| **Total** | **123711** | **28118 (22.7%)** | **95593 (77.3%)** |
