# Supplementary material for: Whole genome sequencing of Plasmodium vivax isolates reveals frequent sequence and structural polymorphisms in erythrocyte binding genes
Source: PLoS Negl Trop Dis. 2020 Oct 12;14(10):e0008234. doi: 10.1371/journal.pntd.0008234 (PMC7581005; doi:10.1371/journal.pntd.0008234)
Supplement: S3 Table — (DOCX) [file pntd.0008234.s003.docx]

**Supplementary Table 3.** Fws statistics for all 44 Ethiopian genomes calculated using the moimix package in R. ^†^ indicates samples with considerably low Fws value and likely polyclonal. For these samples, SNP variants of the major clone were used in genetic analyses.

| **Sample ID** | **Study site** | **Fws Statistic** |
| --- | --- | --- |
| BBH(1)-132 | Jimma | 0.917 |
| BBH(1)-153 | Jimma | 0.722 |
| BBH(1)-162^†^ | Jimma | 0.589^†^ |
| HT(1)-144 | Jimma | 0.905 |
| JHC(2)-100 | Jimma | 0.924 |
| MKH(1)-72 | Jimma | 0.937 |
| SGH(1)-355 | Jimma | 0.917 |
| SGH(1)-357 | Jimma | 0.872 |
| BBH(1)-125 | Jimma | 0.926 |
| SGH(2)-103 | Jimma | 0.910 |
| BBH(1)-137 | Jimma | 0.905 |
| HT(1)-147 | Jimma | 0.893 |
| HT(2)-112 | Jimma | 0.910 |
| JHC(1)-208^†^ | Jimma | 0.512^†^ |
| MKH(2)-71 | Jimma | 0.880 |
| SGH(1)-337 | Jimma | 0.749 |
| SGH(1)-358 | Jimma | 0.924 |
| SGH(1)-359^†^ | Jimma | 0.645^†^ |
| SGH(1)-331 | Jimma | 0.868 |
| SGH(2)-108 | Jimma | 0.917 |
| QS0001-C | Badowacho | 0.904 |
| QS0002-C | Badowacho | 0.931 |
| QS0003-C | Badowacho | 0.923 |
| QS0004-C^†^ | Badowacho | 0.699^†^ |
| QS0011-C^†^ | Hawassa | 0.606^†^ |
| QS0012-C^†^ | Hawassa | 0.552^†^ |
| QS0013-C | Hawassa | 0.901 |
| QS0014-C | Hawassa | 0.900 |
| QS0015-C^†^ | Hawassa | 0.785^†^ |
| QS0016-C | Hawassa | 0.892 |
| QS0018-C | Hawassa | 0.913 |
| QS0025-C | Arbaminch | 0.799 |
| QS0027-C | Arbaminch | 0.926 |
| QS0028-C | Arbaminch | 0.931 |
| QS0031-C^†^ | Arbaminch | 0.423^†^ |
| QS0032-C^†^ | Arbaminch | 0.569^†^ |
| QS0033-C | Arbaminch | 0.920 |
| QS0035-C | Arbaminch | 0.913 |
| QS0037-C | Arbaminch | 0.923 |
| QS0042-C | Halaba | 0.915 |
| QS0044-C | Halaba | 0.889 |
| QS0049-C | Halaba | 0.918 |
| QS0051-C | Badowacho | 0.926 |
| QS0053-C | Badowacho | 0.918 |
