# Supplementary material for: Whole genome sequencing of Plasmodium vivax isolates reveals frequent sequence and structural polymorphisms in erythrocyte binding genes
Source: PLoS Negl Trop Dis. 2020 Oct 12;14(10):e0008234. doi: 10.1371/journal.pntd.0008234 (PMC7581005; doi:10.1371/journal.pntd.0008234)
Supplement: S4 Table — (DOCX) [file pntd.0008234.s004.docx]

**Supplementary Table 4.** Likelihood Ratio Test results of the M1 (neutral model) and M2 models (selection model) in PAML of all the 43 erythrocyte binding gene candidates.

| **Gene ID**  **(PlasmoDB)** | **Gene description** | **d_N_/d_S_ branch average (M1)** | **d_N_/d_S_ branch average (M2)** | **-ln L (M1)** | **-ln L (M2)** |
| --- | --- | --- | --- | --- | --- |
| PVP01_1429700 | duffy binding protein | 0.11 | 1.93 | 6021.82 | 5751.09 |
| PVP01_0613400 | rRNA-processing protein EBP2, putative | 0.84 | 1.36 | 1571.60 | 1568.48 |
| PVP01_0505600 | GPI-anchored micronemal antigen | 0.06 | 0.09 | 2916.95 | 2908.94 |
| PVP01_0824100 | microneme associated antigen, putative | 0.09 | 4.97 | 1531.68 | 1464.36 |
| PVP01_0728900 | merozoite surface protein 1 | 0.08 | 0.32 | 18360.35 | 17082.54 |
| PVP01_0728800 | merozoite surface protein 1 paralog | 0.31 | 0.45 | 12569.98 | 12556.43 |
| PVP01_1031400 | merozoite surface protein 3 | 0.12 | 0.51 | 33768.99 | 31142.80 |
| PVP01_1031300 | merozoite surface protein 3 | 0.11 | 0.43 | 24041.90 | 22168.77 |
| PVP01_1031200 | merozoite surface protein 3 | 0.13 | 0.58 | 28719.11 | 26348.37 |
| PVP01_1031000 | merozoite surface protein 3 | 0.11 | 0.62 | 10922.68 | 9974.29 |
| PVP01_1030900 | merozoite surface protein 3 | 0.14 | 0.36 | 2175.92 | 2113.44 |
| PVP01_1031100 | merozoite surface protein 3 | 0.13 | 0.69 | 3108.202 | 2866.00 |
| PVP01_0418300 | merozoite surface protein 4 | 0.40 | 0.89 | 1416.342 | 1404.35 |
| PVP01_0418400 | merozoite surface protein 5 | 0.14 | 1.06 | 5167.552 | 4740.28 |
| PVP01_1446800 | merozoite surface protein 9 | 0.15 | 1.16 | 5017.59 | 4694.35 |
| PVP01_1129100 | merozoite surface protein 10, putative | 0.35 | 1.16 | 2032.30 | 2027.45 |
| PVP01_0812300 | rhoptry associated adhesin, putative | 1 | 1.26 | 1453.08 | 1452.89 |
| PVP01_0701200 | reticulocyte binding protein 1a | 0.16 | 1.86 | 11958.29 | 11761.89 |
| PVP01_1402400 | reticulocyte binding protein 2a | 0.11 | 1.71 | 10254.66 | 9973.52 |
| PVP01_0800700 | reticulocyte binding protein 2b | 0.13 | 2.40 | 14150.63 | 13824.69 |
| PVP01_0534300 | reticulocyte binding protein 2c | 0.20 | 0.73 | 26607.98 | 25117.34 |
| PVP01_0534400 | reticulocyte binding protein 2 precursor (PvRBP-2), putative | 0.38 | 1.18 | 4607.04 | 4463.34 |
| PVP01_0703800 | high molecular weight rhoptry protein 3, putative | 0.22 | 0.39 | 7966.42 | 7936.76 |
| PVP01_0305300 | rhoptry neck protein 1 | 0.09 | 0.14 | 3971.18 | 3946.69 |
| PVP01_1255000 | rhoptry neck protein 2 | 0.24 | 1.52 | 12348.40 | 12221.49 |
| PVP01_0916600 | rhoptry neck protein 4 | 0.66 | 0.82 | 3578.22 | 3576.57 |
| PVP01_0802200 | transformer-2 protein homolog beta, putative | 0.12 | 0.18 | 4415.19 | 4396.28 |
| PVP01_0202200 | tryptophan-rich protein | 0.16 | 0.5 | 2281.24 | 2247.79 |
| PVP01_0404200 | tryptophan-rich protein | 0.62 | 2.25 | 1362.56 | 1348.87 |
| PVP01_0503400 | tryptophan-rich protein | 1 | 11.98 | 1589.10 | 1580.54 |
| PVP01_0504200 | tryptophan-rich protein | 0.33 | 1.01 | 2188.65 | 2166.87 |
| PVP01_0801800 | tryptophan-rich protein | 0.17 | 0.95 | 4527.37 | 4104.01 |
| PVP01_0948700 | tryptophan-rich protein | 0.11 | 4.07 | 7227.66 | 6603.23 |
| PVP01_0948800 | tryptophan-rich protein | 0.27 | 0.65 | 1802.25 | 1772.00 |
| PVP01_1101400 | tryptophan-rich protein | 0.19 | 1.22 | 2671.76 | 2639.19 |
| PVP01_1201800 | tryptophan-rich protein | 0.11 | 0.26 | 2794.60 | 2760.98 |
| PVP01_1401800 | tryptophan-rich protein | 0.34 | 0.35 | 1195.02 | 1194.95 |
| PVP01_146980 | tryptophan-rich protein | 0.46 | 2.16 | 3428.57 | 3315.71 |
| PVP01_1469900 | tryptophan-rich protein | 0.54 | 2.03 | 1571.85 | 1546.98 |
| PVP01_1470100 | tryptophan-rich protein | 1 | - | 1246.93 | 1246.49 |
| PVP01_0700700 | tryptophan-rich protein | 0.24 | 0.33 | 1308.89 | 1306.92 |
| PVP01_0700800 | tryptophan-rich protein | 0.36 | 1.58 | 1519.23 | 1505.58 |
| PVP01_0503600 | tryptophan-rich protein | 0.41 | 0.74 | 1524.44 | 1518.64 |
