# Supplementary material for: Whole genome sequencing of Plasmodium vivax isolates reveals frequent sequence and structural polymorphisms in erythrocyte binding genes
Source: PLoS Negl Trop Dis. 2020 Oct 12;14(10):e0008234. doi: 10.1371/journal.pntd.0008234 (PMC7581005; doi:10.1371/journal.pntd.0008234)
Supplement: S5 Table — (A) Gene regions that were detected with copy number variation among the 44 Ethiopian P. vivax isolates based on CNVnator. Among them, only two erythrocyte binding gene candidates PvDBP1 and PvMSP3 were detected with high-order copies. (B) Gene regions that showed copy number variation among the 50 Southeast Asian isolates based on CNVnator. Among them, six gene regions were detected with gene duplication and four had high-order copies. (DOCX) [file pntd.0008234.s005.docx]

**Supplementary Table 5.** Gene regions that were detected with copy number variation among the 44 Ethiopian (a) and 50 Southeast Asian (b) *P. vivax* isolates based on CNVnator. Among them, only two erythrocyte binding gene candidates *PvDBP*1 and *PvMSP*3 were detected with high-order copies.

**(a)**

| **Ethiopian *P. vivax*** | |  |  |  |  |
| --- | --- | --- | --- | --- | --- |
| **Chromosome** | **Gene** | **Annotation** | **1 copy** | **2-3 copies** | **4 and higher copies** |
| 6 | PVP01_0623800 | DBP1 | 22 | 19 | 3 |
| 9 | PVP01_0901400 | hypothetical protein | 37 | 7 | 0 |
| 10 | PVP01_1030900 | MSP3.11 | 37 | 6 | 1 |
| 12 | PVP01_1273000 | Plasmodium exported protein, unknown function | 36 | 7 | 1 |
| 12 | PVP01_1247000 | conserved Plasmodium protein, unknown function | 41 | 3 | 0 |
| 14 | PVP01_1470400 | Plasmodium exported protein, unknown function | 41 | 3 | 0 |
| **(b)**  **Southeast Asia *P. vivax*** | |  |  |  |  |
| 6 | PVP01_0623800 | DBP1 | 47 | 3 | 0 |
| 10 | PVP01_1031200 | MSP3.9 | 32 | 17 | 1 |
| 10 | PVP01_1031300 | MSP3.8 | 32 | 17 | 1 |
| 10 | PVP01_1031400 | MSP3.5 | 32 | 17 | 1 |
| 14 | PVP01_1402400 | RBP2a | 48 | 0 | 2 |
| 14 | PVP01_1446800 | MSP9 | 49 | 1 | 0 |
