# Supplementary material for: Whole genome sequencing of Plasmodium vivax isolates reveals frequent sequence and structural polymorphisms in erythrocyte binding genes
Source: PLoS Negl Trop Dis. 2020 Oct 12;14(10):e0008234. doi: 10.1371/journal.pntd.0008234 (PMC7581005; doi:10.1371/journal.pntd.0008234)
Supplement: S6 Table — (DOCX) [file pntd.0008234.s006.docx]

**Supplementary Table 6.** *F_ST_* values calculated between study sites using the VCFTools procedure.

| Study site | Arbaminch | Halaba | Hawassa | Badowacho |
| --- | --- | --- | --- | --- |
| Arbaminch | - | - | - | - |
| Halaba | -0.020 | - | - | - |
| Hawassa | 0.017 | 0.006 | - | - |
| Badowacho | -0.009 | -0.041 | 0.013 | - |
| Jimma | 0.229 | 0.327 | 0.259 | 0.239 |
