# Supplementary figures and images for: Whole genome sequencing of Plasmodium vivax isolates reveals frequent sequence and structural polymorphisms in erythrocyte binding genes
Source: PLoS Negl Trop Dis. 2020 Oct 12;14(10):e0008234. doi: 10.1371/journal.pntd.0008234 (PMC7581005; doi:10.1371/journal.pntd.0008234)

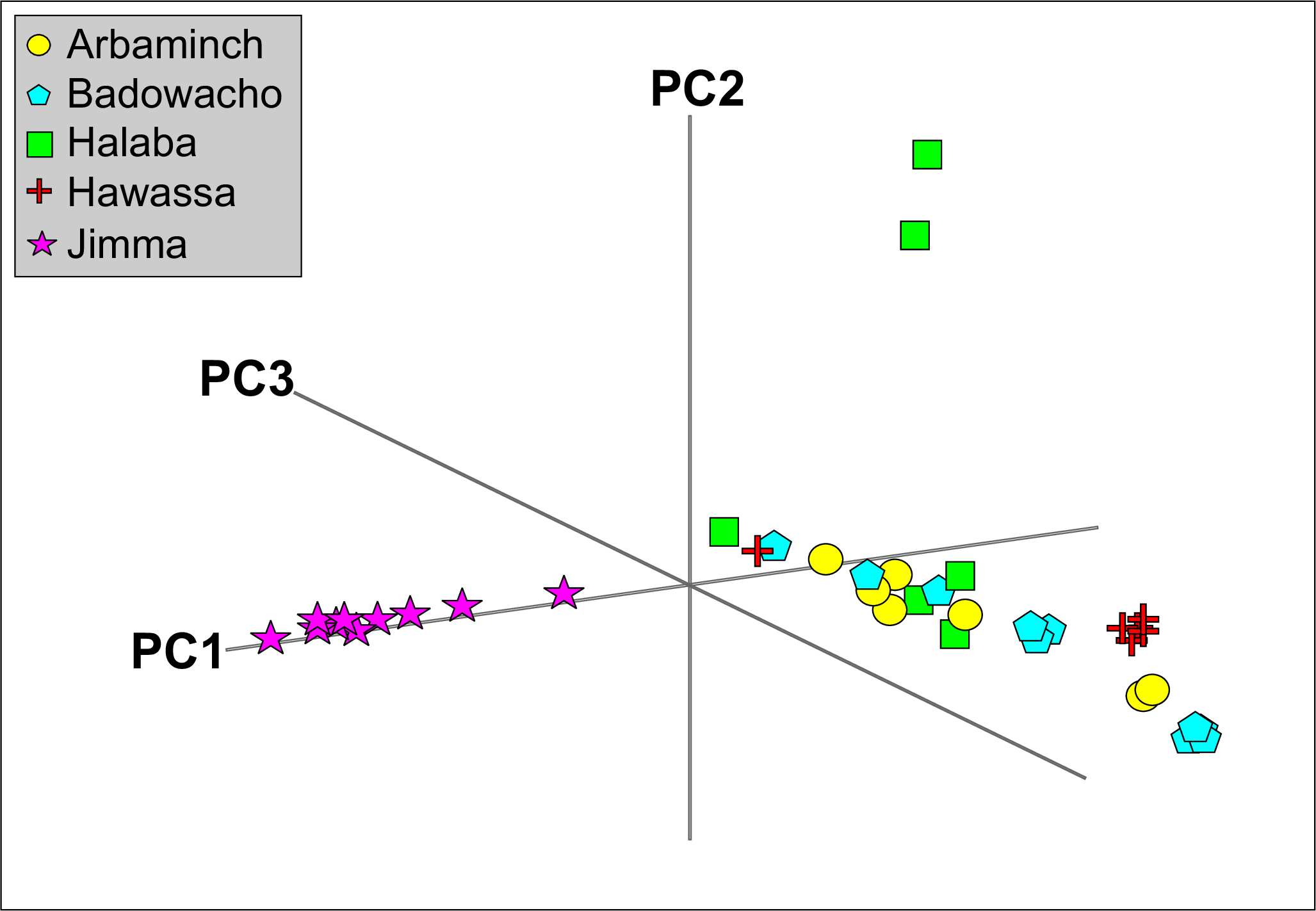

Supplement: S1 Fig — Samples obtained from Jimma were clustered together, whereas samples from Arbaminch, Badowacho, Hawassa, and Halaba were mixed, with the exception of two samples from Hawassa. This clustering pattern suggested that there was considerable genetic variation among study sites even at a small geographical scale. (TIF) [file pntd.0008234.s007.tif]
