# Supplementary material for: Whole genome sequencing of Plasmodium vivax isolates reveals frequent sequence and structural polymorphisms in erythrocyte binding genes
Source: PLoS Negl Trop Dis. 2020 Oct 12;14(10):e0008234. doi: 10.1371/journal.pntd.0008234 (PMC7581005; doi:10.1371/journal.pntd.0008234)
Supplement: Attachment — Submitted filename: Responses to reviewers_final.docx [file pntd.0008234.s008.docx]

Anthony Ford

Woodward Hall 381C

University of North Carolina at Charlotte

Email: aford29@uncc.edu

Office: 704-687-8532

Lab: 704-687-1926

July 31, 2020

Dear Editor,

We submit the revised version of the manuscript entitled “Whole Genome Sequencing of Plasmodium vivax isolates reveals frequent sequence and structural polymorphisms in Erythrocyte binding genes”. We are thankful to the reviewers’ constructive comments/suggestions and have taken all comments to improve this manuscript. We believe that this manuscript is scientifically valid and technically sound. In this revised version, we address the reviewers’ queries point-by-point. All changes can be viewed by track-changes in the uploaded word file. Our responses to the reviewers’ comments are detailed below in this response letter (reviewers’ comments in italic and our responses in bold).

We sincerely look forward to receiving your decision on this revised manuscript.

Yours Sincerely,

Anthony Ford

*Reviewer's Responses to Questions

Methods*

*Reviewer #1: The methods and analysis as they are, are sufficient to understand what experiments and computational work was done. But many details are missing that make the conclusions difficult to understand.

1. The read depth from all genomes seems in conflict with the analyses done. Line 234 says a read depth of >30 was required for all SNPs, but Table 1 indicates about 22 samples with an average read depth less than 30.*

**Response:**

**Our response: Table 1 shows an overall coverage of the genomes and did not indicate the filtering on the read depth. We identify SNPs based on post variant call (P12 L269).**

*2. I'm not quite sure how clonality was determined. I don't see the results of the microsatellite experiments. Additionally, were the publicly available data from monoclonal infections? If they weren't then many of these analyses might be difficult to understand.

Reviewer #2: (No Response)*

**Our Response: In order to determine the clonality of the 44 samples we used the R package moimix to calculate the Fws statistics. We explained the approach in Methods (P11 L245) and results (P19 L417; Supplementary Table 3).**

*--------------------

Results
Reviewer #1: I think these studies are missing a critical outgroup of vivax genomes from a different area. (eg, among the genes that are under positive selection, are they just under positive selection in this dataset from Ethiopia, or is it observed elsewhere?) Additionally, are there differences in SNPs between Duffy-negative and Duffy-positive patients?*

**Our response: We have compared the results of positive selection with other geographical samples based on previous studies including Pearson et al. 2016, Hupalo et al. 2016, and Auburn et al. 2018 (P26-P27 L573-L608).**

**We are in the process of obtaining more Duffy-negative samples/genomes so that we can compare with the SNPs detected in Duffy-positives. This paper mainly aims to provide genome characteristics of the Ethiopian *P. vivax* as baseline information and transmission pattern among study sites. We are not yet able to present the differences between Duffy negative and Duffy positive patients until we obtain sufficient samples that can provide meaningful and strong statistical power.** *What is the explanation for the DBP CNV is different geographic locations? If I am understanding correctly, Fig 6 shows relatively low transmission between sites, yet multiple DBP copies are found in a few different sites? Can the associated SNPs explain how this came about?*

**Our Response: We included 50 P. vivax genomes from Southeast Asia (Cambodia and Thailand) as an outgroup to compare the copy numbers we see in Ethiopia**. **The number of published genomes from Cambodia and Thailand is comparable to the sample size of our Ethiopia samples. Based on our analyses and previous findings, DBP copies ranges from 1 to 2-3 copies in the Southeast Asian isolates, despite a higher transmission intensity than Ethiopia. Thus, DBP copies may not be related to transmission intensity but host-parasite interaction during the invasion process. Given that Duffy phenotype is more diverse among the Ethiopians than Asians (Lo et al. PLoS NTD 2019), it is possible that P. vivax expands DBP to multiple copies to enhance binding affinity and invasion to different host phenotype. Different patterns of gene flow among geographical locations may determine the spread and distribution of high-DBP copies strains. Also, P. vivax with high-DBP copies could be evolved from single-copy DBP multiple times independently at different geographic locations, as suggested in our phylogenetic analyses*.* (P25)**
 *Line 334 - These numbers seem significant, but shouldn't the size of the chromosome be taken into account? Maybe some statistics (expected # if SNPs were randomly distributed)*

**Our response: We have commented on the size of the chromosome and the number of SNPs detected. They do not appear to be dependent on one another (P16 L385).**

*Line 347 - What does the lowest number mean? Among all annotated genes, or just the erythrocyte binding genes?*

**Our response: This was done only on the 43 erythrocyte binding protein genes instead of all gene regions (P18 L386).**

*Table 1 - Samples generated in this study should be indicated in some way.
Figure 4 - I think this should include gene names.
Figure 5 - I think where these samples came from is an important part of the interpretation of this figure and is missing.
Figure 6 - I don't think a 3D PCA is required here.

Reviewer #2: (No Response)*

**Our Response: We have updated the plots/figures to address these concerns as follow. Genomes generated in this study are labeled in Table 1. Gene names in Figure 4 are presented in Supplementary Table 5. We have indicated the sample ID and corresponding location for each branch in Figure 5. The PCA plot is removed from Fig. 6 and presented as Supplementary Figure 1.**
 *--------------------

Conclusions
Reviewer #1: I think the conclusions are supported by the data, but it is difficult to determine if the gene flow/evolution studies are unique to Ethiopia from this work as is. And I think a greater discussion of how this data can be used to understand Duffy-negative infections is missing.*

***Our Response:* We are in the process of obtaining more Duffy-negative samples/genomes so that we can compare with the SNPs detected in Duffy-positives. In this paper, we aim to provide genome characteristics of the Ethiopian *P. vivax* as baseline information and transmission pattern among study sites. We are not yet able to present the differences between Duffy negative and Duffy positive patients until we obtain sufficient samples that can provide meaningful and strong statistical power.**

*Line 484 - How did you validate these genes? I think this is an important point because as is, the entire focus of the manuscript is on evolution in genes thought to be related to invasion, so this validation is important.*

**Our response: The erythrocyte binding genes were selected based on the literature documentation and their functions are currently validated by ongoing binding assay. We have also checked the function of each gene using multiple databases, resulting in a panel of 43 erythrocyte binding genes (P26 L570).**

*Line 497 - Are there SNPs between the copy numbers? ie are all DBPs in the same genome identical?*

**Our response: Based on Gunalan et al. 2016 and Lo et al. 2019, the DBP tandem copies had identical sequences (P25 L551).**
 *Reviewer #2: (No Response)

--------------------

Editorial and Data Presentation Modifications?
Reviewer #1: 116 - This sentence needs editing
123 - These genes should all be in a consistent PvP01 gene name

Reviewer #2: (No Response)*

**Our response: We have checked and edited the gene names to ensure consistency (P7 L149-150).**

*--------------------

Summary and General Comments*

*Reviewer #1: The results presented here do not match the hypothesis or question initially introduced. The introduction sets up the paper as being about SNPs or CNVs related to vivax malaria among Duffy-negative individuals. And choosing this region of Ethiopia, with increasing rates of vivax malaria and Duffy-negative individuals, makes this kind of genomic study very exciting and an important dataset. But this question is not addressed in any of the analyses.*

***Our response:* In this paper, we aim to provide genome characteristics of the Ethiopian *P. vivax* as baseline information and transmission pattern among study sites. We are in the process of obtaining more Duffy-negative samples/genomes so that we can compare with the SNPs detected in Duffy-positives. We are not yet able to present the differences between Duffy negative and Duffy positive patients until we obtain sufficient samples that can provide meaningful and strong statistical power.**

*Going forward, two possible analyses could be performed to better place the data within the initial question. 1) Add an analysis where the Duffy-positive and Duffy-negative individuals are compared. It's possible though that this analysis would be too difficult and beyond the scope of the paper because A) 24 of the publicly available samples are unknown in regards to Duffy, B) because transmission in this region, Duffy-negative and Duffy-positive patients may still have active transmission between each other, which would be confounding. So an alternative analysis would be: 2) Add an analysis with other vivax genomes from other regions. I understand this is similar to "Auburn et al 2019," but it is difficult to interpret these results without knowing how these vivax compare to vivax in other regions where Duffy-negative infections are not happening.*

**Our Response: We included 50 P. vivax genomes from Southeast Asia (Cambodia and Thailand) as an outgroup to compare the SNP and copy number variants with the Ethiopian P. vivax (NEW Figs 3 and 4)**.

*Reviewer #2: The manuscript described 20 new P. vivax genome sequences from Ethiopia and performed population genomics analysis of these samples with 24 samples from the same region published earlier. While the overall study appears to be sound, more emphasis may need to be on the populations in Ethiopia, and whether more detailed SNP analysis from WGS provides better resolution of the parasite populations in this country than the studies done using microsatellite markers. If this is true, a panel of SNPs that can be used to better reflect parasite population divisions in this country would indeed be very useful. However, just from reading the abstract, these important issues are not reflected in the abstract.*

**Our Response: Thank you for this suggestion. We are currently working on constructing a panel of high-performance SNPs that can be provide high resolution to population divisions of P. vivax in Ethiopia. We will test the utility of these SNPs with broader samples in our ongoing study (P29 L657-L659).**

*Major comments
1.      Authors claimed that these Pv sequences “all allowed us to develop a panel of informative Single Nucleotide Polymorphic markers diagnostic at a micro-geographical scale”. This would be a nice to identify a panel of SNPs that can be used to distinguish parasites at micro- scale. However, I did not see anywhere about this panel of SNPs. This would be interesting given the divergence of Jimma parasites from other parts.*

**Our Response: We are currently working on constructing a panel of high-performance SNPs that can be provide high resolution to population divisions of P. vivax in Ethiopia. We will test the utility of these SNPs with broader samples in our ongoing study (P29 L657-L659).**

*2.      The authors detect clonality using 7 microsattelite markers, then claim regarding the WGS “Similarly, we concluded that the percentage of the P. vivax reads covered in the additional 24 samples were high enough to reflect the dominant signal of the variants and negate polyclonal influences.” From what I could tell there’s no discussion of how they came to this conclusion. Analyses such as non-reference allele distributions and Est-MOI are very easy to perform with WGS data and would confirm clonality.*

**Our response: We analyze our genomes with MoiMix to determine Fws metric that was used for inbreeding analysis to inform clonality (see Methods (P11 L245); Results (P19 L417); and Supplementary Table 3).**

*3.      Bowtie was used to analyze the 22 samples and call for SNPs, whereas BWA-MEMv.2 was used to align the 24. I wonder why not doing all alignments using the same program, since two programs with different parameter settings may give you different results. Could this be the cause of the significant divergence of Jimma samples from the rest since SNP calling used different methods?*

**Our response: We clarify that all genomes were aligned with BWA-MEMv.2 using the same default settings (P11 L232).**

*4.      Strainhub appears to use a relatively new methodology which uses phylogenetic tree state changes to map closeness. It would be good to use additional methods to confirm some of the findings regarding population sharing and geneflow; i.e. IBD sharing could help with high/low transmission regions, Fst could be used to complement genetic isolation findings. Additionally, I’d like to hear the authors comment on whether or not their analysis is sensitive to sample size, as the authors of the programs commented in their paper that “the results are only as strong as the underlying phylogenetic data (sampling across metadata states and taxa…)” and this could factor into their identification of Halaba, which only has 3 samples, as a sink. This makes us feel that the source-sink analysis is not robust.*

**Our Response: We have performed the analysis and included Fst values calculated between study sites using the vcftools software and Plink (P17 L365; P22 L493; Supplementary Table 6).**

*5.      I suggest that the authors use additional tests such as Structure to compare the relationships among the parasites from different regions. Together with phylogeny and PCA, this may provide further corroboration of the data.*

**Our response: We have analyzed the genetic structure of the genomes with Admixture and provided a comparison of genetic composition between the regions (P17 L370; P22 L497; New Fig 7)**

*6.      I think the authors try to analyze the RBC invasion related genes in order to identify genes potentially involved in the invasion of Duffy negative RBC. However, this part is flawed from the use of genes that are apparently involved in RNA metabolism. The authors analyzed a DBP family of genes because these genes carry an abbreviation of DBP. Two of the genes included in this family are the Duffy binding protein (DBP), whereas the rest are simply proteins of the DEAD box RNA helicases – DEAD box protein (which happen to be abbreviated as DBP). But they have nothing to do with invasion.*

**Our response: Thank you for this comment and we have removed all DBP-helicase genes throughout.**

*7.      The pir gene copy variation part may not be correct and undermines their conclusions. PIR genes are really common sources of mismapping. I don’t see any specific actions to control for this in the data. On line 458 authors suggest that using P01 mitigates this because its subtelomeric ends are more complete, but to my understanding a lot of the reason mismapping occurs on these genes is because the repeat regions are highly repetitive and the genes are highly variable, meaning it’s very easy for algorithms to misidentify the best match. Because of the high coverage variability this could affect copy number predictions from CNVnator. Additionally, because of misaligned reads it could affect SNP-calling—a problem that can also come from duplications. It may be worth doing a supplemental analysis specifically on high interest PIR genes to confirm mismapping wasn’t a problem either using read visualization or PCR. This may similarly to other claims of the msp3 gene family amplifications.*

**Our response: We have trimmed the chromosome ends and removed hypervariable regions as mentioned in Pearson et al 2016. We have also removed the PIR CNV’s from the figures and throughout the manuscript.**

*Minor comments:
Sample collection part is confusing. Line 184-196: This part described collection of 22 Pv clinical samples in 2016 and DNA extracted for sequencing. Then they further described in lines 197-201 that another larger set of samples were genotyped and used for sequencing. I am confused how many from each place or sample set? The line 203, “The original 22 samples were processed to remove reads other than P. vivax”, which original, the 22 samples from Jimma in 2016? How about the larger set of samples? Then they extracted WGS from additional 24 samples – please add a reference showing that these are previously published data.*

**Our response: We clarify the information of the samples including 22 new Ethiopian genome, 24 published Ethiopian genomes, and 50 Southeast Asian published genomes analyzed in this study (P11).**

*Line 208: “the Duffy status of each of these 24 samples is not known”. I assume from this statement that the rest of 22 samples have known Duffy status. If this is the case, would that be useful to compare the parasite isolates from Duffy negative with those from Duffy positive hosts (even though the number is small)?*

**Our response: In this paper, we aim to provide genome characteristics of the Ethiopian *P. vivax* as baseline information and transmission pattern among study sites. We are in the process of obtaining more Duffy-negative samples/genomes so that we can compare with the SNPs detected in Duffy-positives. We are not yet able to present the differences between Duffy negative and Duffy positive patients until we obtain sufficient samples that can provide meaningful and strong statistical power.**

*Line 186: malaria symptomatic (who……..), to me this is too early since you collected blood for microscopy (next sentence). For this sentence, I would use those with symptoms suspected of malaria infection.*

**Our response: We correct the sentence as suggested (P10 L209).**

*Line 227: “3,000 million” > 3000? 3million? 3billion?*

**Our response: We have this as 3 billion (P12 L261).**

*Line 343: I wonder if the authors provide somewhere backgrounds for msp3 gene family (how these new nomenclatures correspond to many references describing msp3alpha, beta and gamma)?*

**Our response: We have described the background and function of the MSP3 gene family in the context of the observed signal of selection (P26 L587).**

*Line 379 (and elsewhere): the members included are not all DBP family proteins.*

**Our response: Thank you for this comment and we have removed all DBP-helicase genes throughout.**

*Line 386-390: Pir copy number part needs to be removed (see reasons above)*

**Our response: We have removed the PIR genes throughout the manuscript as stated above.**

*Line 392: duplications were observed for msp3 on chromosome 10. Do you consider all the msp3 gene family members as duplications? Or a particular member. Actually many msp3 gene members are suspected to have arisen from past duplication events and then divergence, since their sequences differ dramatically.*

**Our response: We have checked and specified the duplications detected in MSP3.11 (P21). We provide further background of the MSP3 gene family on P26.**

*Figure 6A is useful and may be better to include collection sites information. Figure 6B-D do not seem to provide more information.*

**Our response: We have indicated the sample ID and location for each branch in Figure 6.**

*Line 411: “could have expanded multiply among samples at different locations.” > could have resulted from multiple duplication events*

**Our response: Thank you for this comment and we have corrected this sentence (P22 L487).**

*Line 429: I’m not sure it’s a good idea to compare to reference 19 for blanket number of SNPs because you used two different pipelines to call them.*

**Our response: We have edited the description of SNPs among geographical regions (P24).**

*Line 441: “remarkably high diversity in RBP2 than in RBP1” > remarkably high diversity in RBP2 compared to RBP1*

**Our response: This sentence has been removed due to substantial changes in the text.**

*Line 423-424: Given the size of Ethiopia and the close proximity of the collection sites, it is hard to understand why the lack of gene flow? This deserves some discussion (any gene flow barriers, different ethnicities? …….)*

**Our response: We have provided possible explanation to the genetic differences observed between Jimma and other sites (P29 L645).**
